# Transcriptomic Profile of Canine DH82 Macrophages Infected by *Leishmania infantum* Promastigotes with Different Virulence Behavior

**DOI:** 10.3390/ijms23031466

**Published:** 2022-01-27

**Authors:** Alicia Mas, Abel Martínez-Rodrigo, Javier Carrión, José Antonio Orden, Juan F. Alzate, Gustavo Domínguez-Bernal, Pilar Horcajo

**Affiliations:** 1 INMIVET, Department of Animal Health, Faculty of Veterinary Science, Complutense University of Madrid, 28040 Madrid, Spain; alimas@ucm.es (A.M.); abelmr@ucm.es (A.M.-R.); javier.carrion@ucm.es (J.C.); jaorden@vet.ucm.es (J.A.O.); 2 Centro Nacional de Secuenciación Genómica-CNSG, Facultad de Medicina, Departamento de Microbiología y Parasitología, Universidad de Antioquia, Medellín 050010, Colombia; jfernando.alzate@udea.edu.co; 3 Animal Health and Zoonoses (SALUVET) Group, Animal Health Department, Faculty of Veterinary Sciences, Complutense University of Madrid, 28040 Madrid, Spain; phorcajo@ucm.es

**Keywords:** *Leishmania infantum*, virulence, canine macrophage, RNA-seq, DH82 cells

## Abstract

Zoonotic visceral leishmaniosis caused by *Leishmania infantum* is an endemic disease in the Mediterranean Basin affecting mainly humans and dogs, the main reservoir. The leishmaniosis outbreak declared in the Community of Madrid (Spain) led to a significant increase in human disease incidence without enhancing canine leishmaniosis prevalence, suggesting a better adaptation of the outbreak’s isolates by other host species. One of the isolates obtained in the focus, IPER/ES/2012/BOS1FL1 (BOS1FL1), has previously demonstrated a different phenotype than the reference strain MCAN/ES/1996/BCN150 (BCN150), characterized by a lower infectivity when interacting with canine macrophages. Nevertheless, not enough changes in the cell defensive response were found to support their different behavior. Thus, we decided to investigate the molecular mechanisms involved in the interaction of both parasites with DH82 canine macrophages by studying their transcriptomic profiles developed after infection using RNA sequencing. The results showed a common regulation induced by both parasites in the phosphoinositide-3-kinase–protein kinase B/Akt and NOD-like receptor signaling pathways. However, other pathways, such as phagocytosis and signal transduction, including tumor necrosis factor, mitogen-activated kinases and nuclear factor-κB, were only regulated after infection with BOS1FL1. These differences could contribute to the reduced infection ability of the outbreak isolates in canine cells. Our results open a new avenue to investigate the true role of adaptation of *L. infantum* isolates in their interaction with their different hosts.

## 1. Introduction

Leishmaniases are parasitic diseases caused by different species of the genus *Leishmania*, which are transmitted via female phlebotomine sand flies [1]. In the Mediterranean Basin, zoonotic visceral leishmaniosis (ZVL) due to *Leishmania infantum* is considered endemic and constitutes a major public health challenge, as it affects both humans and dogs, the main reservoir [2]. Traditionally, its prevalence in the human population was low, and it was linked to immunosuppression states [3]. However, in 2009, an important human leishmaniosis outbreak was declared in the Community of Madrid (Spain), resulting in a dramatic increase in its incidence, with more than 700 cases in this region alone until 2016 [4]. This outbreak has attracted the interest of the scientific community, as it presents interesting singularities. Although the etiological agent was *L. infantum*, a high number of immunocompetent patients were affected [5], but when investigating the responsible isolates, no new emerging genotype of this parasite was found [6]. In addition, seroprevalence and xenodiagnostic studies confirmed the establishment of a mainly sylvatic transmission cycle, independent of canids [7], with lagomorphs as alternative reservoirs for the parasite [8,9].

The interaction between *Leishmania* spp. and host immune system cells is decisive for the development of the disease [10]. Macrophages are one of the most important target cells for this parasite, playing a key role in facilitating both the control and progression of infection [11]. Furthermore, the parasite has its own mechanisms to evade the host immune response, adapting to the intracellular environment to survive and multiply [12]. Thus, *Leishmania* encodes virulence factors that allow it to interfere with host immune cells, affecting the activity of various surface receptors and different signaling events, such as the mitogen-activated kinase (MAPK) pathway, the Janus kinase/signal transducer and activator of transcription (JAK-STAT) pathway or the phosphoinositide-3-kinase–protein kinase B/Akt (PI3K-Akt) pathway [13,14]. Parasite modulation of these pathways affects the synthesis of inflammatory cytokines and the production of leishmanicidal molecules [14,15,16].

In previous works, our group investigated the reasons behind the modifications in the ecoepidemiological features of the human leishmaniosis outbreak of Madrid, describing a higher virulence profile of the BOS1FL1 (IPER/ES/2012/BOS1FL1) *L. infantum* isolate obtained from the focus when compared with the well-characterized strain BCN150 (MCAN/ES/96/BCN150), first *in vitro* [17] and then using an *in vivo* murine model of visceral leishmaniosis (VL) [18]. This increase in the virulence of the outbreak isolate could be related to the high number of cases found in the human population, but it has not been reflected in an increase in canine leishmaniosis (CanL) prevalence. In this context, we investigated the interaction of BCN150 and BOS1FL1 with canine monocytes and macrophages derived from peripheral blood [19]. Contrary to what we observed in mice, BOS1FL1 showed a lower capacity of infection than BCN150 in canine monocytes and macrophages. However, the mechanisms involved in this difference were not unravel [19]. Therefore, in the present work, we studied the interaction of *L. infantum* BCN150 and BOS1FL1 promastigotes with the canine macrophage cell line DH82 by RNA sequencing (RNA-seq) with the aim of detecting transcriptomic differences that could shed light on the different behavior of these parasites in canine cells.

## 2. Results and Discussion

### 2.1. Study Design and Infection Dynamics

The *L. infantum* BOS1FL1 isolate, obtained from the human leishmaniasis outbreak of Madrid, exhibited different infection ability depending on the host species when compared to the reference strain BCN150. In the murine VL model, BOS1FL1 showed a higher virulence profile than the reference strain in terms of dissemination in target organs and modulation of the host immune response [18]. In contrast, when infecting primary canine monocytes and macrophages, BCN150 showed the highest infection capacity. Nevertheless, when defensive mechanisms of dog macrophages were studied, differences that explain the change in infectivity between parasites were not found [19]. To further study the interaction of BCN150 and BOS1FL1 with cells of canine origin, we decided to investigate the global transcriptional response to the infection by performing an RNA-seq assay. RNA-seq techniques have been used in recent years to analyze the transcriptome profile induced by infection with several *Leishmania* species in cells of different origins [20]. However, to the best of the authors’ knowledge, no previous studies have used RNA-seq to analyze the interaction of *L. infantum* with cells of canine origin, and it is difficult to translocate the miscellaneous results obtained with other host and parasite species to comprehensively describe the *Leishmania*-infected canine macrophage response.

**Figure 1:**
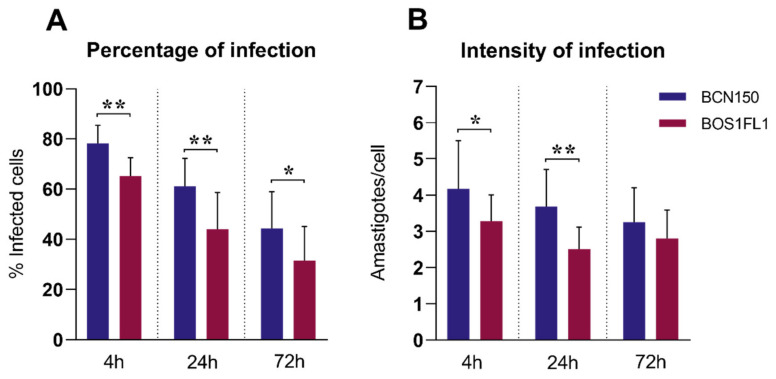
*Leishmania infantum* BCN150 and BOS1FL1 infection capacity in canine DH82 macrophages. DH82 cells were infected with *L. infantum* BCN150 or BOS1FL1 at a ratio of 5:1 parasites:cells. The percentage of infected cells (**A**) and the mean number of amastigotes per infected cell (intensity of infection) (**B**) were determined at 4, 24 and 72 h post-infection (pi). Data (mean ± SD) from four independent experiments are shown. Statistical analysis was performed with a parametric Student’s *t* test using GraphPad Prism software version 8.30. Asterisks (*) indicate statistically significant differences (* *p* < 0.05, ** *p* < 0.01).

In this work, we employed the canine macrophage cell line DH82, with the aim of improving the reproducibility of the assay and minimizing the biological diversity using primary cells obtained from different donors [21,22]. Thus, we first analyzed the infectivity of BCN150 and BOS1FL1 in DH82 cells to rule out any differences compared to the behavior exhibited by these parasites after infection of primary canine macrophages. DH82 cells were exposed to stationary promastigotes of both parasites, and the percentage and intensity of infection were determined at 4 h, 24 h and 72 h post-infection (pi). In agreement with the results obtained in primary cells [19], the percentage (Figure 1A) and the intensity of infection (Figure 1B) were higher with BCN150 than BOS1FL1, although the difference in intensity at 72 h was not significant. The infection index, in concordance with the above parameters, was significantly lower in DH82 macrophages infected with BOS1FL1 than in those infected with BCN150 at all time points (Table 1). These results replicated those achieved in primary canine monocytes and macrophages and were in contrast with the results reported in murine cells [19], suggesting a differential behavior of these parasites in their interaction with host cells depending on their species.

### 2.2. Global Transcriptome Analyses

We decided to characterize the global transcriptional profiles of both parasites and DH82 cells after 4 h of interaction. This time point was chosen because previous studies found an increase in the response of murine and human macrophages infected with *Leishmania* spp. at 4 h, with a gradual decrease in the number of differentially expressed genes (DEGs) up to 72 h [23,24]. Furthermore, our results revealed that at 4 h pi, DH82 cells showed the highest percentages of infection with both BOS1FL1 and BCN150.

**Figure 2:**
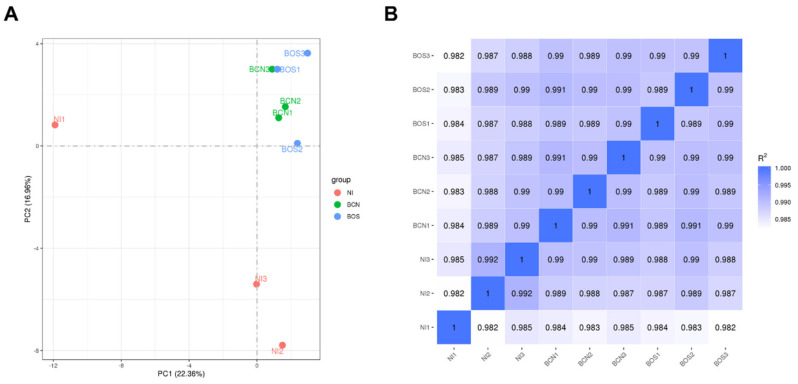
Correlation analysis in DH82 *L. infantum*-infected and uninfected samples. Principal component analysis (PCA) (**A**) was performed to observe variation between samples. The percentage variability captured by the two principal components is displayed across PC1 and PC2 represented on the X and Y axes. Reproducibility between RNA sequencing data replicates (**B**) was evaluated based on the Pearson correlation coefficient according to all gene expression levels (fragments per kilobase of transcript per million mapped reads, FPKM) of each sample.

RNA-seq analysis was performed for three independent replicates of each condition: noninfected cells (NI), BCN150-infected cells (BCN) and BOS1FL1-infected cells (BOS). Approximately 770 million raw reads were generated, ranging between 80 and 90 million per sample, which were further processed to obtain clean read data (Supplementary Table S1). The mean percentages of reads mapped to the dog genome were 96% for uninfected cells and 79% for infected cells (Supplementary Table S2). After exclusion of reads from the dog genome, the remaining reads were mapped to the *L. infantum* genome, obtaining between 6.8 and 8.5 million mapped reads in each sample (Supplementary Table S3). To study the distribution of data and reproducibility between samples under the same conditions, correlation analysis and principal component analysis (PCA) were carried out. The results revealed a correct reliability of biological replicates but also a similar distribution between BOS1FL1- and BCN150-infected samples, suggesting a close gene expression pattern in these groups (Figure 2).

### 2.3. Infection with L. infantum BCN150 and BOS1FL1 Induces Poor Regulation of the DH82 Canine Macrophage Gene Expression Profile

**Figure 3:**
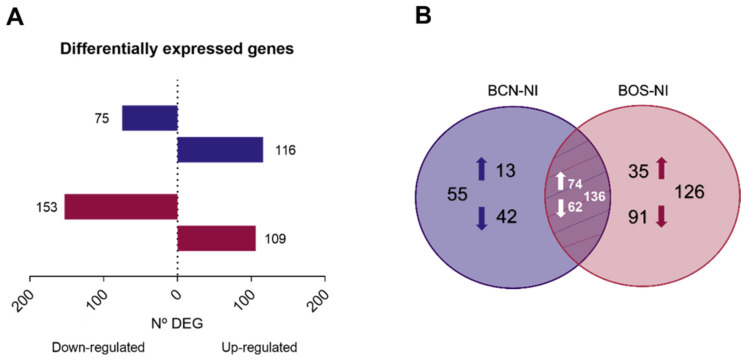
Differentially expressed genes in DH82 cells infected with *L. infantum* BCN150 and BOS1FL1 versus noninfected cells for 4 h. The total number of differentially expressed genes (DEGs) (**A**) in *L. infantum*-infected macrophages relative to uninfected controls is represented as horizontal bar plots. Genes were considered DEGs when they presented a false discovery rate value (FDR) ≤ 0.05. Box lengths depict the numbers of genes down-regulated and up-regulated. (**B**) Venn diagram showing the number of unique and shared DEGs in BCN-NI and BOS-NI comparisons. The results were obtained from three biological replicates for each condition.

To understand the molecular mechanisms involved in the response of DH82 cells to infection with BCN150 and BOS1FL1, a differential expression analysis between infected and uninfected cells was performed. The highest differences were found in the BOS-NI comparison with 262 DEGs. Among these, 109 were up-regulated and 153 were down-regulated. On the other hand, 191 DEGs were found in the BCN-NI comparison, of which 116 were up-regulated and 75 were down-regulated (Figure 3A, Supplementary Tables S4 and S5). Interestingly, when we investigated the similarity degree of all these DEGs, we found that 136 genes were DEGs in both comparisons, while 126 and 55 genes were exclusive to BOS1FL1 and BCN150 infection, respectively (Figure 3B). Thus, although we obtained an infection percentage close to 80% after 4 h, the number of DEGs observed in infected cells indicated that both BCN150 and BOS1FL1 induced poor regulation of the DH82 gene expression profile.

Our results contrasted with those from Dillon et al., 2015, who observed higher transcriptomic changes (with more than 6500 DEGs) when comparing *Leishmania major*-infected human macrophages with uninfected cells at 4 h pi, even though the infection rates were very similar to ours [24]. Discrepancies with our results could be associated with parasite species and the origin of the cells used in the research. Indeed, a recent RNA-seq study conducted by Andreu et al., 2017, compared the response of the murine macrophage cell line J774 with murine bone marrow-derived macrophages to the infection with *Mycobacterium tuberculosis* and primary cells were more highly reactive. Among other possibilities, the authors suggested that the tumor origin of J774 cells could influence their activation status, favoring a lower response to infection than primary cells [25]. Nevertheless, tumor cell lines have previously been used in RNA-seq studies. More specifically, DH82 cells have been employed to explore their interaction with canine distemper virus, finding important variations in gene expression patterns after infection [26], although their use with *Leishmania* has not been described to date. In the case of *L. infantum*, another assay performed in 2020 used the human monocyte cell line THP-1 to analyze inflammasome activation and in agreement with our results, the authors described a poor response of cell line to the infection [27]. However, it should be noted that the study was performed at 8 h pi, perhaps at 4 h pi the results would have been different.

The regulation induced in the transcriptome profile of DH82 macrophages infected by BCN150 and BOS1FL1 could, therefore, be conditioned by their tumoral nature, but also by a possible silent entry of the parasite into the cells, avoiding their defensive response. These data support the hypothesis that not only the pathogen but also the origin and cell type in which the studies are carried out could condition the results obtained, making their interpretation more difficult and adding more complexity to the understanding of the host-pathogen interaction process.

### 2.4. BCN150 and BOS1FL1 Induce a Specific Modulation of the DH82 Canine Macrophage Transcriptomic Response

**Figure 4:**
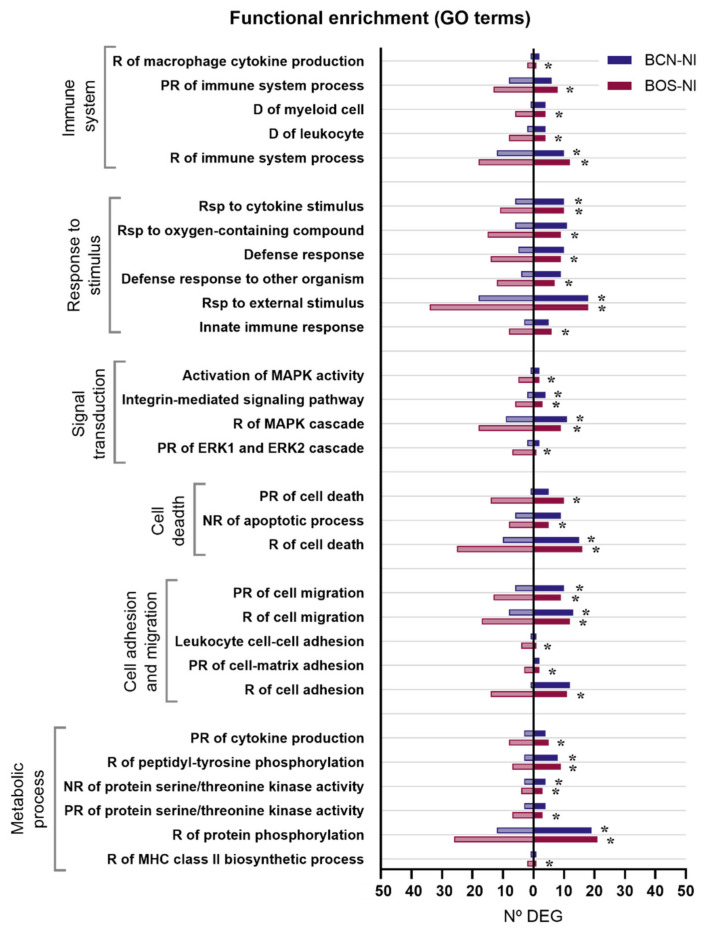
Gene ontology (GO) terms enriched in DEGs between DH82 cells infected with *L. infantum* BCN150 and BOS1FL1 and noninfected cells. The graph shows the most relevant GO terms enriched from DEGs in the BCN-NI and BOS-NI comparisons. The x-axis represents the number of DEGs associated with each GO term. Dark bars indicate up-regulated genes and light bars indicate down-regulated genes. Asterisks indicate enriched GO terms considered statistically significant based on an FDR value ≤ 0.05. R, regulation; PR, positive regulation; NR, negative regulation; D, differentiation; Rsp, response.

When comparing the gene expression patterns developed in DH82 canine macrophages after BCN150 and BOS1FL1 infection, a specific modulation according to the strain or isolate was observed, with some DEGs found exclusively in cells infected with each parasite. To further explore the different responses triggered, we performed Gene Ontology (GO), related to biological processes, [28] and Kyoto Encyclopedia of Genes and Genomes (KEGG) [29] enrichment analyses using DEGs from BCN-NI and BOS-NI. Thus, the study of BCN-NI comparison resulted in 118 enriched GO terms grouped into 28 clusters (Supplementary Table S6). In the case of the BOS-NI comparison, the modulation exerted on the cellular response was greater, with 326 enriched GO terms grouped into 48 clusters (Supplementary Table S7). In both cases, terms related to the regulation of the immune system, response to stimuli, signal transduction, apoptosis, metabolism, cell adhesion and migration were obtained (Figure 4).

In agreement with the GO terms, the KEGG enrichment analysis revealed 9 statistically significant pathways for BCN-NI (Supplementary Table S8) and 39 statistically significant pathways for BOS-NI (Supplementary Table S9). Notably, all pathways enriched in BCN150-infected canine macrophages were also enriched in BOS1FL1-infected cells, while the outbreak isolate modulated some specific pathways that could play key roles in the observed behavioral differences (Table 2 and Table 3).

**Figure 5:**
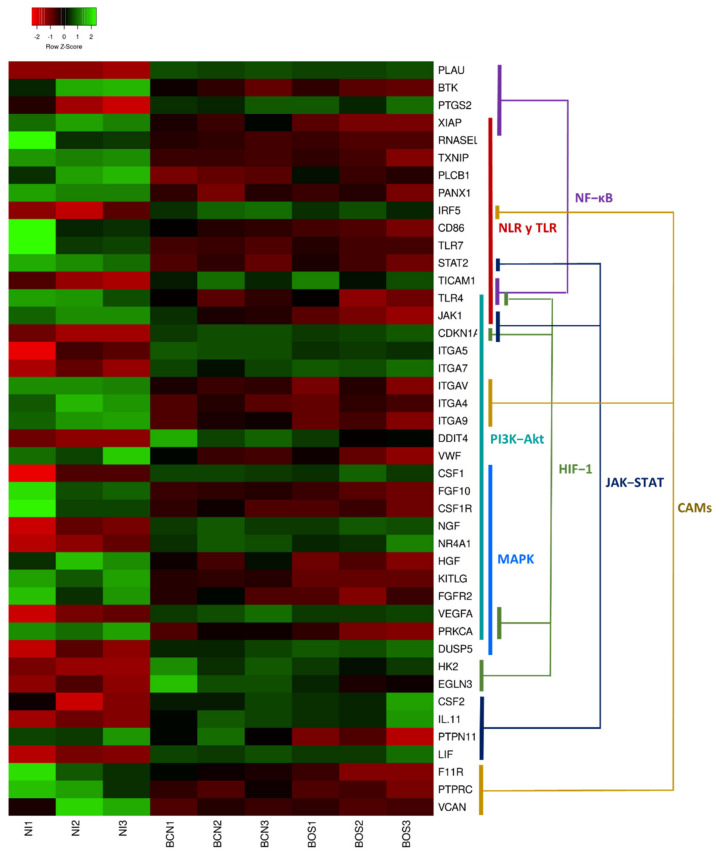
Heatmap of differentially expressed genes in canine DH82 macrophages. The graph represents a selection of DEGs showing row Z-scores based on expression data of three replicates of DH82 cells infected with *L. infantum* BCN150 (BCN1–BCN3) and BOS1FL1 (BOS1–BOS3) and noninfected cells (NI1–NI3). Colored segments show the pathways in which DEGs are involved. The heatmap was generated using Heatmapper (http://www2.heatmapper.ca, accessed on 16 June 2021).

Figure 5 graphically represents the expression levels of DEGs involved in the most relevant pathways. Regarding common pathways, the PI3K-Akt and NOD-like receptor (NLR) signaling pathways were found to be among those most related to the cellular response to infection. When we analyzed exclusive BOS-NI modulation, we identified pathways related to pathogen recognition (Toll-like receptor-TLR signaling pathway), phagocytosis (phagosome) and signal transduction, such as tumor necrosis factor (TNF), MAPK, JAK-STAT and nuclear factor-κB (NF-κB). All these signaling cascades are involved in mediating important cellular processes such as proliferation, survival, cell death and activation of immune defense [15,16,30,31]. It is widely described that one of the most commonly employed mechanisms by *Leishmania* spp. to subvert the host immune system consists of the manipulation of these pathways that are directly related to the synthesis of inflammatory cytokines and leishmanicidal compounds such as nitric oxide (NO) or reactive oxygen species (ROS) [13]. Moreover, when we investigated genes implicated in KEGG pathways enriched exclusively in the BOS-NI comparison, we found some interesting DEGs that appeared only in BOS1FL1-infected DH82 cells. For instance, we found the up-regulated gene *CSF2* which encodes granulocyte-macrophage colony-stimulating factor (GM-CSF), which is related to leukocyte activation and the down-regulated gene *PKRCA* which encodes protein kinase C (PKC)-α. Some studies have demonstrated that *Leishmania* can impair PKC-dependent respiratory burst activity and NO production in macrophages, increasing its intracellular survival [32]. However, PKC activity is also implicated in the activation of the extracellular signal-regulated kinase (ERK)-MAPK cascade [33], which plays an important role in IL-10 production, an anti-inflammatory cytokine related to parasite survival [16].

Accordingly, with these data, we have already reported that BCN150- and BOS1FL1-infected primary canine macrophages in coculture with lymphocytes exhibited a different cytokine production profile as determined by ELISA, dominated by IL-10 (favoring susceptibility) or IFN-γ (favoring resistance), respectively [19]. Altogether, these findings suggest that BOS1FL1 induced a different regulation than BCN150 in canine macrophages, affecting important pathways related to the immune system, which could impact the ability of this isolate to establish infection in dogs.

### 2.5. Infection of DH82 Cells with BCN150 or BOS1FL1 Affects PI3K-Akt and NLR Signaling Pathway Regulation with Some Critical Differences

**Figure 6:**
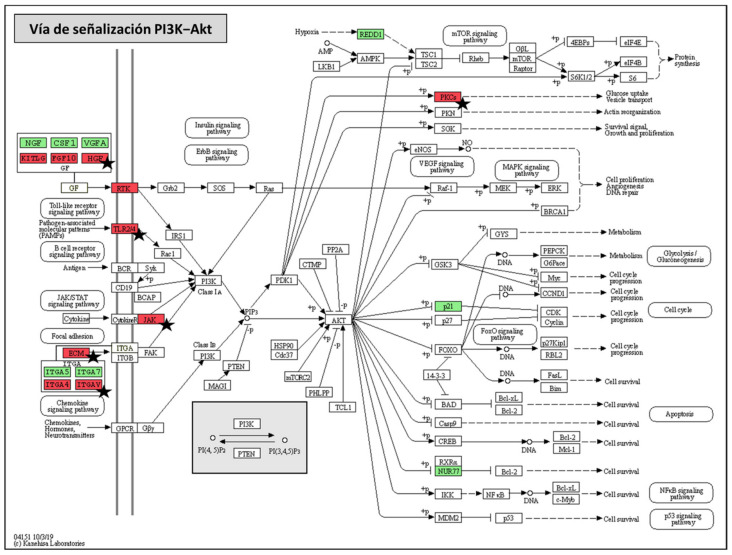
Phosphoinositide-3-kinase–protein kinase B/Akt (PI3K-Akt) KEGG pathway map. Graphic representation of PI3K-Akt signaling pathway (modified based on KEGG database) in which DEGs that were found in both BCN150-infected cells and BOS1FL1-infected cells with respect to the control are highlighted in green (up-regulated genes) and red (down-regulated genes). Black stars indicate those genes found exclusively in BOS1FL1-infected cells.

The PI3K-Akt signaling pathway was found to be the most strongly modulated pathway in DH82 canine macrophages after infection with *L. infantum* BCN150 and BOS1FL1, with the highest number of DEGs involved. Our results revealed 8 up-regulated genes (*ITGA5, ITGA7, NGF, CSF1, VEGFA, NR4A1, CDKN1A* and *DDIT4*) that appeared in both BCN-NI and BOS-NI comparisons and 12 down-regulated genes, 4 common (*ITGA4, KITLG, CSFR1* and *FGF10*) and the remaining 8 only differentially expressed in BOS1FL1-infected cells (*FGR2, HGF, PRKCA, ITGAV, VWF, JAK1* and *TLR4*) (Figure 6).

By studying the resulting products of these genes, we found different growth factors, integral membrane proteins such as integrins, and cell surface receptors such as TLR and tyrosine kinase. All these products contribute to the activation of the PI3K enzyme [34]. Although both over- and underexpressed genes were found in BCN-NI and BOS-NI, the number of down-regulated genes induced by BOS1FL1 was significantly higher. This pathway plays an important role in the host response to *Leishmania* infection [14]. PI3K enzyme activity has been associated with the progression of leishmaniosis by modulating the phagocytosis capacity of macrophages and the secretion of anti-inflammatory cytokines [35,36]. In addition, some studies describe that PI3K/Akt activation following *Leishmania amazonensis* infection may promote a decrease in the expression of the enzyme inducible nitric oxide synthase (iNOS) and consequently in NO production [37]. Our work reveals that the PI3K-Akt signaling pathway was the most affected when comparing DH82-infected and uninfected cells, which could be associated with a determinant function of this pathway in the canine macrophage response against *L. infantum*. Furthermore, the increased number of down-regulated genes induced by BOS1FL1 compared to BCN150 could lead to reduced activation of PI3K-AKT in response to infection with this isolate, contributing to its limited infectivity.

Our results showed that the DNA damage-inducible transcript 4 (*DDIT4*) gene (also known as *REDD1*) was overexpressed in BCN-NI and BOS-NI comparisons. *DDIT4* encodes the synthesis of an inhibitory protein of mammalian target of rapamycin (mTOR) complex 1 (mTORC1) [38], a downstream effector of PI3K/AKT implicated in cell proliferation, survival and the immune response [39]. Studies on the role of mTOR in the host response to *Leishmania* are still scarce, and their results are controversial. Some authors have reported that mTOR inhibition by rapamycin has a protective effect against *L. donovani,* avoiding the decrease in IL-12 and the increase in IL-10 production induced by the parasite [14,40]. In contrast, other studies have demonstrated that *L. major* is able to inactivate mTORC1 dampening eukaryotic translation initiation factor 4E (eIF4E)-binding protein 1 (4E-BP1) phosphorylation, which is required for eIF4E activation. The result is the inhibition of host cell protein synthesis and iNOS activation, promoting parasite persistence in the phagosome [41]. The up-regulation of *DDIT4* in DH82-infected cells, which was even higher in the cells infected with BCN150 than in those infected with BOS1FL1, could therefore result in the inhibition of mTOR1, promoting parasite intracellular growth. However, further studies are needed to investigate the mechanisms employed by *L. infantum* to modulate *DDIT4* and the mTOR1 complex and their effects on parasite virulence and the canine host defense response.

Immune system cells have receptors that are indispensable in pathogen recognition, such as TLR and NLR, which play a key role in the development of the immune response [42]. It has been widely described that these receptors can exert a defensive function in leishmaniosis [43], contributing to the production of inflammatory cytokines by macrophages [44]. Our results showed that *L. infantum* BCN150 and BOS1FL1 triggered differences in the regulation of TLR and NLR signaling pathways. The gene encoding the TLR7 receptor was underexpressed in both the BCN-NI and BOS-NI comparisons, while the gene encoding the TLR4 receptor was exclusively down-regulated in BOS-NI. Some studies have shown that TLR4 activation contributes to the control of *L. major* infection in mice [45], but the role this receptor plays in canine leishmaniosis is unclear, with both increased and decreased expression levels observed in different organs and tissues of *L. infantum*-infected dogs [46,47]. Information regarding the role of the TLR7 receptor in leishmaniosis is also scarce, but the use of TLR7 agonists has been associated with protective phenotypes against infection [48,49]. The NLRP3 receptor, a member of the NLR family, directly influences inflammasome activation and, together with NF-κβ pathway activation, leads to the synthesis of proinflammatory cytokines [50]. The infection of DH82 cells with BCN150 and BOS1FL1 resulted in some down-regulated DEGs, such as *PANX1*, *TXNIP, RNASEL* and others exclusive from BOS1FL1 (*TLR4, XIAP*) or BCN150 (*PLCB1*) related to the activation of both NLRP3 and NF-κβ [51,52,53,54,55]. Some authors suggest that *Leishmania* can modulate the host defensive response by inhibiting NLRP3 receptor activation to ensure its survival [56,57,58].

Interestingly, the interaction between NLRP3 and mTOR has been described previously. Rojas Márquez et al., 2018, reported that mTOR inhibition during *Trypanosoma cruzi* infection induced NLRP3 activation in murine cells to control parasite replication [59]. In contrast, Moon et al., 2015, demonstrated that mTORC1-induced glycolysis provided a critical mechanism for NLRP3 inflammasome activation in macrophages [60]. Our results suggest that both BCN150 and BOS1FL1 could attempt to evade the defensive response of canine DH82 macrophages by synergistically modulating mTOR and NLRP3 activation. These results highlight the need for further knowledge of the role that these receptors play in the establishment of infection by different *L. infantum* strains or isolates in their interaction with canine hosts.

Overall, the transcriptomic profiling of DH82-infected cells indicates that BCN150 and BOS1FL1 could interfere with the macrophage defense machinery by simultaneously affecting the expression of different signal transduction events. This modulation could also be more advantageous for the survival of BCN150, which could contribute to the differential behavior of both parasites.

### 2.6. BCN150 and BOS1FL1 Displayed Similar Transcriptome Profiles after Their Interaction with Canine DH82 Cells

The gene expression profiles of BCN150 and BOS1FL1 after interaction with DH82 cells were compared. We identified 8300 *L. infantum* genes, of which 8270 were expressed in both parasites, while 18 and 12 were exclusively expressed in BOS1FL1 and BCN150, respectively. Genes that were not common, encoded, mostly, different transfer RNA (tRNA) molecules and hypothetical proteins of unknown function. Differential expression analysis revealed only 23 DEGs between BCN150 and BOS1FL1. Of these, 15 were overexpressed in BCN150, mainly related to energy metabolism, intracellular transport, cell cycle or transcription. The remaining 8 genes were overexpressed in BOS1FL1, related to the synthesis of the 40S and 60S ribosomal proteins and histones H1, H2A and H4 (Supplementary Table S10).

Although kinetoplastid parasites rely almost exclusively on posttranscriptional gene regulation, RNA-seq techniques have been used before to reveal interesting features of *Leishmania spp*. transcriptome under different conditions [61]. The success of this protozoa in establishing infection in mammalian hosts depends on its ability to differentiate into amastigotes and survive in the phagosomes of infected cells [62]. Interestingly, among the genes overexpressed in BCN150, we found the gene encoding the synthesis of the heat shock protein HSP83, which constitutes the cytoplasmic form of the HSP90 protein involved in parasite signaling and differentiation into amastigotes [63]. Conversely, the gene encoding histone H1 was overexpressed in the BOS1FL1 isolate. This protein has been linked to a reduced ability of promastigotes to differentiate into amastigotes and a decreased infectivity of the parasites *in vitro* and *in vivo* [64]. These data point to the possibility that the differential behavior exhibited by both parasites could be due to the sum effects of multiple factors involved in the parasite-host interaction.

## 3. Materials and Methods

### 3.1. Cell and Parasite Culture

The canine macrophage cell line DH82 (ATCC^®^-CRL-10389^TM^), was cultured in Dulbecco’s modified Eagle’s medium (DMEM) (Thermo Fisher Scientific, Waltham, MA, USA) supplemented with 10% heat-inactivated fetal bovine serum (FBS) (Thermo Fisher Scientific, Waltham, MA, USA), 100 U/mL penicillin and 100 μg/mL streptomycin (Lonza, Basel, Switzerland) at 37 °C and 5% CO_2_. The *L. infantum* BOS1FL1 (IPER/ES/2012/BOS1FL1) isolate, obtained from *Phlebotomus perniciosus* captured in the leishmaniosis outbreak of Madrid [65], was compared to the well-characterized strain BCN150 (MCAN/ES/96/BCN150), which has been used before in other studies of pathogen-host interactions [18,19,66,67]. Both parasites were first inoculated into BALB/c mice to maintain their virulence [68]. Five weeks after mouse infection, promastigotes were obtained and grown at 26 °C in Schneider’s medium (Dominique Dutscher, Issy-les-Moulineaux, France) supplemented with 20% heat-inactivated FBS (Thermo Fisher Scientific, Waltham, MA, USA), 200 U/mL penicillin and 200 µg/mL streptomycin (Lonza, Basel, Switzerland).

### 3.2. Macrophage Infection

DH82 cells (1 × 10^6^ per well) were seeded in six-well plates and incubated overnight at 37 °C and 5% CO_2_. The infection assays were performed with stationary phase promastigotes of BOS1FL1 and BCN150 for 4 h at 37 °C using a ratio of 5 parasites per macrophage as previously described [19]. Subsequently, cells were washed 3 times with phosphate-buffered saline (PBS) (Lonza, Basel, Switzerland) to remove the extracellular parasites and recovered by scraping followed by centrifugation for 10 min at 1350 g. Cells were then employed for RNA isolation. Parasite infectivity was evaluated in parallel and under the same conditions, by infecting 2 × 10^5^ cells per well seeded on sterile 13-mm coverslips in 24-well plates. The infection was carried out with the same ratio of 5:1 parasite: cells for 4 h, 24 h and 72 h. The percentage of infected cells and the mean number of amastigotes per infected cell (defined as the intensity of infection) were determined by counting 400 cells in duplicate after Giemsa staining under an Olympus BX41 optical microscope. The infection index was calculated by multiplication of both parameters as previously described [69].

### 3.3. RNA Isolation, cDNA Library Construction and Sequencing

Total RNA was isolated from 3 independent replicates of DH82 macrophages from 3 different conditions: uninfected, infected with BCN150 and infected with BOS1FL1, using the NZY Total RNA Isolation Kit (NZYTech, Lisbon, Portugal) following the manufacturer’s instructions. RNA concentration and quality were determined using a spectrophotometer at A260/A280 (Nanodrop ND1000, Thermo Scientific, Waltham, MA, USA). Additionally, RNA integrity was assessed via electrophoresis on a 1% agarose gel stained with GelRed (Biotium, Fremont, CA, USA) and using the RNA Nano 6000 Assay Kit of the Bioanalyzer 2100 system (Agilent Technologies, Santa Clara, CA, USA). All samples had an RNA integrity number (RIN) between 8.7 and 9.9. A total amount of 1 μg of RNA per sample was used as input material for the RNA sample preparations. Sequencing libraries were generated using the NEBNext^®^ UltraTM RNA Library Prep Kit for Illumina^®^ (NEB, Ipswich, MA, USA) following the manufacturer’s recommendations. Briefly, mRNA was purified from total RNA using poly-T oligo-attached magnetic beads, and cDNA fragments were synthesized using DNA Polymerase I and RNase H. After the adenylation of the 3′ ends, NEBNext Adaptors were ligated to prepare for hybridization. To select 150- to 200-bp cDNA fragments, the libraries were purified with the AMPure XP system (Beckman Coulter, Brea, CA, USA). Then, the obtained products were enriched using polymerase chain reaction (PCR) and library quality was assessed on the Agilent Bioanalyzer 2100 system. Finally, the library preparations were sequenced on an Illumina platform and paired-end reads were generated. Preparation of the RNA library and transcriptome sequencing were conducted by Novogene Co., Ltd. (Cambridge, UK).

### 3.4. Quality Control, Mapping to the Reference Genome and Data Normalization

Quality assessment of the raw data was performed using the FASTP tool (http://github.com/OpenGene/fastp, accessed on 9 October 2020) [70]. Clean data were obtained by removing reads containing adapters, poly-N sequences and reads with low quality (Q score < 5). Q20, Q30 [71] and GC content [72] were calculated. Paired-end clean reads from each sample were aligned to *Canis lupus familiaris* genome version CanFam3.1 (NCBI: GCA_ 000002285.2) and *Leishmania infantum JPCM5* genome version ASM287v2 (NCBI: GCA_000002875.2) obtained from the ENSEMBL/NCBI database [73,74] using HISAT2 software (https://ccb.jhu.edu/software/hisat2/inde, accessed on 9 October 2020 [75]. The gene expression levels were estimated by transcript abundance, which was calculated as the fragments per kilobase of transcript per million mapped reads (FPKM) for each gene, including known and novel genes, by normalizing for gene length and sequencing depth [76].

### 3.5. Differential Expression and Functional Enrichment Analyses

First, correlations between samples of the same condition were evaluated for their acceptance as biological replicates and PCA analysis was carried out to visualize the relationship between all different conditions [77]. Next, differential expression analysis between groups was performed by the DESeq2 R package (http://www.r-project.org, accessed on 9 October 2020) using a model based on a negative binomial distribution [78]. The resulting *p*-value were adjusted using Benjamini and Hochberg’s [79] approach for controlling the FDR value. Genes with adjusted *p*-value  <  0.05 and |log2(FoldChange)|  >  0 were considered DEGs. Gene Ontology enrichment analysis of DEGs was used for semantic clustering. Enriched GO terms were identified by the statistical overrepresentation test using PANTHER 16.0 (http://www.pantherdb.org, accessed on 15 June 2021) [80,81,82]. To facilitate their analysis, REVIGO was used to identify nonredundant GO terms. All the analyses were performed with an allowed similarity of 0.5 and with default settings in advanced options [83]. Enriched biological pathways were identified using the KEGG database [29,84,85] and STRING 10 version 11.0 (https://www.string-db.org, accessed on 15 June 2021) [86]. GO terms and KEGG pathways with FDR < 0.05 were considered statistically significant. Finally, functional enrichment analysis of the *L. infantum* DEGs list was performed using the TriTrypDB database (https://tritrypdb.org, VEuPathDB, accessed on 15 June 2021) [87].

## 4. Conclusions

Our study is the first RNA-seq-based analysis of the interaction between *L. infantum* and canine cells. Our results reveal that the infection of DH82 macrophages with BCN150 and BOS1FL1 induced modulation of the transcriptomic response affecting some important pathways related to the immune response such as the PI3K-Akt or NOD-like receptor signaling cascades. Additionally, some differences were found when comparing the transcriptomes of cells infected by each parasite, pointing to a strain/isolate-specific regulation of the macrophage gene expression profile. These differences could be related to the decreased infection capacity of BOS1FL1 in comparison with BCN150 in canine cells and they could have contributed to the adaptation of these isolates from Madrid’s leishmaniosis outbreak to other hosts, such as hares and rabbits. These findings provide insights to further investigate the mechanisms involved in leishmaniosis immunopathogenesis in canids and to decipher the true role of the adaptation and coevolution of different *L. infantum* isolates in the virulence and pathogenic process resulting from the interaction of this protozoan with its different hosts.

## Figures and Tables

**Table 1:** *L. infantum* BCN150 and BOS1FL2 infection index in canine DH82 cells.

Infection Index	DH82	
	BCN150	BOS1FL1
4 h pi	330 ± 128 **	214 ± 58
24 h pi	222 ± 96 **	111 ± 64
72 h pi	163 ± 98 *	101 ± 63

DH82 cells were infected with *L. infantum* BCN150 or BOS1FL1 at a ratio of 5:1 parasites:cells for 4 h, 24 h and 72 h. The infection index was calculated by multiplying the percentage of infected cells and the mean number of amastigotes per infected cell (intensity of infection). Data (mean ± SD) from four independent experiments are shown. Statistical analysis was performed with a parametric Student’s *t* test using GraphPad Prism software version 8.3.0. Asterisks (*) indicate statistically significant differences (* *p* < 0.05, ** *p* < 0.01).

**Table 2:** The most relevant KEGG pathways enriched in BCN-NI.

KEGG Pathway	Number of DEG	Pathway Size	FDR
PI3K-Akt signaling pathway	12	319	0.0156
VEGF signaling pathway ^a^	4	51	0.0216
NOD-like receptor signaling pathway ^b^	5	129	0.0165
Rap1 signaling pathway	9	193	0.0156

^a^ Only up-regulated DEGs were used as input. ^b^ Only down-regulated DEGs were used as input.

**Table 3:** The most relevant KEGG pathways enriched in BOS-NI.

KEGG Pathway	Number of DEG	Pathway Size	FDR
PI3K-Akt signaling pathway	20	319	0.0000048
VEGF signaling pathway	5	51	0.0084
HIF-1 signaling pathway	6	94	0.0161
TNF signaling pathway ^a^	4	99	0.0323
Cytokine-cytokine receptor interaction	9	208	0.0174
NOD-like receptor signaling pathway	8	129	0.0061
Rap1 signaling pathway	11	193	0.0022
Ras signaling pathway	11	212	0.0032
Toll-like receptor signaling pathway	5	79	0.0334
JAK-STAT signaling pathway	7	136	0.0207
NF-κappa B signaling pathway	6	84	0.0109
MAPK signaling pathway	12	273	0.0061
Focal adhesion	12	184	0.00098
Phagosome	6	125	0.0485
Cell adhesion molecules (CAMs)	8	126	0.0061
ECM-receptor interaction	9	76	0.0084

^a^ Only up-regulated DEGs were used as input.

## Data Availability

The raw sequencing data generated during this study are openly available in the Sequence Read Archive (SRA) at the National Center for Biotechnology Information (NCBI) servers under the Bio-Project PRJNA701880, with the following accession numbers: SAMN17915430, SAMN17915429 and SAMN17915428. https://dataview.ncbi.nlm.nih.gov/object/PRJNA701880?reviewer=nq9jlh8sni6igpo062n722rhj9.

## Supplementary Materials

The following are available online at https://www.mdpi.com/article/10.3390/ijms23031466/s1.
