# Supplementary material for: Transcriptomic Profile of Canine DH82 Macrophages Infected by Leishmania infantum Promastigotes with Different Virulence Behavior
Source: Int J Mol Sci. 2022 Jan 27;23(3):1466. doi: 10.3390/ijms23031466 (PMC8835757; doi:10.3390/ijms23031466)
Supplement: Supplementary file 1 [file ijms-23-01466-s001.zip › Supplementary Table S1.pdf]

International Journal of
Molecular Sciences

MDPI

Supplementary Table S1. RNA-seq data quality summary

Sample	Raw reads	Clean reads	Error rate (%)	Q20(%)	Q30 (%)	GC (%)
**NI R1**	43175966	42803376	0.02	98.03	94.52	50.00
**NI R2**	42524308	42178430	0.02	98.05	94.53	49.04
**NI R3**	47038553	46626622	0.02	98.01	94.39	49.58
**BCN R1**	45166943	44550696	0.02	97.92	94.34	51.69
**BCN R2**	47368713	46867870	0.03	97.87	94.21	51.88
**BCN R3**	41440103	41044095	0.03	97.88	94.23	51.80
**BOS R1**	43741209	43249902	0.02	97.91	94.30	51.41
**BOS R2**	40854656	40368080	0.02	97.99	94.49	51.50
**BOS R3**	41435550	40850676	0.02	98.05	94.65	51.56
