# Supplementary material for: Transcriptomic Profile of Canine DH82 Macrophages Infected by Leishmania infantum Promastigotes with Different Virulence Behavior
Source: Int J Mol Sci. 2022 Jan 27;23(3):1466. doi: 10.3390/ijms23031466 (PMC8835757; doi:10.3390/ijms23031466)
Supplement: Supplementary file 1 [file ijms-23-01466-s001.zip › Supplementary Table S2.pdf]

International Journal of *Molecular Sciences*

MDPI

Supplementary Table S2. Read alignment to the *Canis lupus familiaris* genome

Sample	Total reads	Total mapped reads	Mapping rate (%)
**NI R1**	85606752	81804853	95.56
**NI R2**	84356860	80920542	95.93
**NI R3**	93253244	89230524	95.69
**BCN R1**	89101392	70090761	78.66
**BCN R2**	93735740	73992850	78.94
**BCN R3**	82088190	64195954	78.20
**BOS R1**	86499804	68713256	79.44
**BOS R2**	80736160	64485975	79.87
**BOS R3**	81701352	64312991	78.72
