# Supplementary material for: Transcriptomic Profile of Canine DH82 Macrophages Infected by Leishmania infantum Promastigotes with Different Virulence Behavior
Source: Int J Mol Sci. 2022 Jan 27;23(3):1466. doi: 10.3390/ijms23031466 (PMC8835757; doi:10.3390/ijms23031466)
Supplement: Supplementary file 1 [file ijms-23-01466-s001.zip › Supplementary Table S3.pdf]

International Journal of
Molecular Sciences

MDPI

Supplementary Table S3. Read alignment to the *Leishmania infantum* genome

Sample	Total reads	Total mapped reads	Mapping rate (%)
**BCN R1**	21413648	8182463	38.21
**BCN R2**	22326356	8500058	38.07
**BCN R3**	20144002	7761730	38.53
**BOS R1**	20096416	7566644	37.65
**BOS R2**	18457056	6840244	37.06
**BOS R3**	19497644	7480189	38.36
